# Supplementary material for: How medical education survives and evolves during COVID-19: Our experience and future direction
Source: PLoS One. 2020 Dec 18;15(12):e0243958. doi: 10.1371/journal.pone.0243958 (PMC7748283; doi:10.1371/journal.pone.0243958)
Supplement: S1 Fig — (PPTX) [file pone.0243958.s001.pptx]

## Slide 1
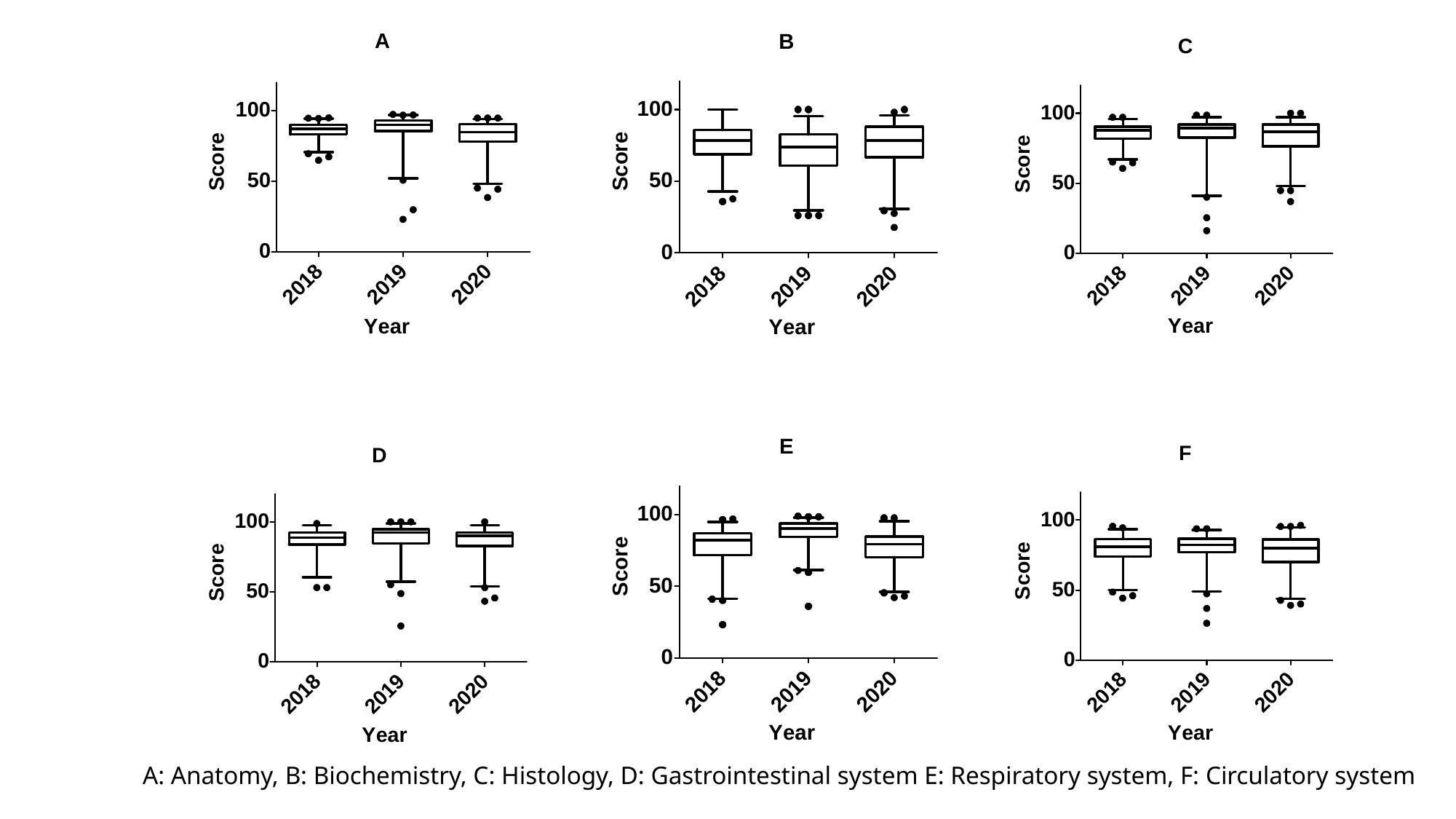

A: Anatomy, B: Biochemistry, C: Histology, D: Gastrointestinal system E: Respiratory system, F: Circulatory system
