# Supplementary material for: How medical education survives and evolves during COVID-19: Our experience and future direction
Source: PLoS One. 2020 Dec 18;15(12):e0243958. doi: 10.1371/journal.pone.0243958 (PMC7748283; doi:10.1371/journal.pone.0243958)
Supplement: S1 Table — (DOCX) [file pone.0243958.s002.docx]

**S1 Table. Students’ examination scores in 2020 compared to in 2018 and 2019**

| **Least Squares Means Estimate** | | | | | | |
| --- | --- | --- | --- | --- | --- | --- |
|  |  |  |  | **95% CI** | |  |
| **course** | **year** | **Estimate** | **SE** | **Lower** | **Upper** | **p-value** |
| Anatomy | **2018** | 85.9489 | 0.9289 | 84.1272 | 87.7705 |  |
|  | **2019** | 87.3822 | 0.9303 | 85.5577 | 89.2068 |  |
|  | **means (2018, 2019)** | 86.6656 | 0.6668 | 85.3578 | 87.9733 |  |
|  | **2020** | 82.5483 | 0.9597 | 80.6662 | 84.4304 |  |
|  | **2020 vs (2018, 2019)** | -4.1173 | 1.1503 | -6.3731 | -1.8615 | 0.0004 |
| Biochemistry | **2018** | 79.8207 | 0.9319 | 77.9930 | 81.6483 |  |
|  | **2019** | 70.6685 | 0.9186 | 68.8669 | 72.4701 |  |
|  | **means (2018, 2019)** | 75.2446 | 0.6633 | 73.9437 | 76.5454 |  |
|  | **2020** | 73.6151 | 0.9320 | 71.7873 | 75.4429 |  |
|  | **2020 vs (2018, 2019)** | -1.6295 | 0.9703 | -3.5324 | 0.2734 | 0.0932 |
| Histology | **2018** | 86.3060 | 0.9231 | 84.4956 | 88.1164 |  |
|  | **2019** | 85.3602 | 0.9152 | 83.5653 | 87.1551 |  |
|  | **means (2018, 2019)** | 85.8331 | 0.6611 | 84.5366 | 87.1296 |  |
|  | **2020** | 84.0597 | 0.9571 | 82.1827 | 85.9367 |  |
|  | **2020 vs (2018, 2019)** | -1.7734 | 1.1405 | -4.0101 | 0.4633 | 0.1201 |
| Gastrointestinal system | **2018** | 86.2435 | 0.9461 | 84.3880 | 88.0990 |  |
|  | **2019** | 88.4833 | 0.9249 | 86.6695 | 90.2971 |  |
|  | **means (2018, 2019)** | 87.3634 | 0.6687 | 86.0520 | 88.6749 |  |
|  | **2020** | 85.5667 | 0.9347 | 83.7337 | 87.3997 |  |
|  | **2020 vs (2018, 2019)** | -1.7967 | 1.1370 | -4.0265 | 0.4330 | 0.1142 |
| Respiratory system | **2018** | 78.4883 | 0.9428 | 76.6394 | 80.3372 |  |
|  | **2019** | 88.8710 | 0.9104 | 87.0856 | 90.6564 |  |
|  | **means (2018, 2019)** | 83.6797 | 0.6645 | 82.3764 | 84.9829 |  |
|  | **2020** | 76.5601 | 0.9347 | 74.7271 | 78.3931 |  |
|  | **2020 vs (2018, 2019)** | -7.1195 | 1.1335 | -9.3424 | -4.8967 | <0.0001 |
| Circulatory system | **2018** | 78.7914 | 0.9461 | 76.9359 | 80.6469 |  |
|  | **2019** | 80.6844 | 0.9137 | 78.8926 | 82.4763 |  |
|  | **means (2018, 2019)** | 79.7379 | 0.6654 | 78.4329 | 81.0429 |  |
|  | **2020** | 76.9426 | 0.9347 | 75.1096 | 78.7756 |  |
|  | **2020 vs (2018, 2019)** | -2.7953 | 1.1340 | -5.0192 | -0.5715 | 0.0138 |
