# Supplementary material for: How medical education survives and evolves during COVID-19: Our experience and future direction
Source: PLoS One. 2020 Dec 18;15(12):e0243958. doi: 10.1371/journal.pone.0243958 (PMC7748283; doi:10.1371/journal.pone.0243958)
Supplement: S1 Appendix — (DOCX) [file pone.0243958.s004.docx]

**S1 Appendix. Survey Form**

**Student Course Evaluation Form**

■ Please answer each question using 5-point Likert scale

Rating system: 5: strongly agree, 4: agree, 3: neither agree nor disagree, 2: disagree, and 1: strongly disagree.

| Overall satisfaction on the course | | |
| --- | --- | --- |
| 1 | I am generally satisfied with the course | ⑤ ④ ③ ② ① |
| 2 | The educational objectives of the course were clearly presented | ⑤ ④ ③ ② ① |
| 3 | The course lectures were well-organized in relation to each other | ⑤ ④ ③ ② ① |
| 4 | I am generally satisfied with the volume of learning | ⑤ ④ ③ ② ① |
| Technical aspects of online lectures | | |
| 5 | I am generally satisfied with the progress of the online lecture | ⑤ ④ ③ ② ① |
| 6 | I am generally satisfied with the video quality of the lecture | ⑤ ④ ③ ② ① |
| 7 | I am generally satisfied with the sound quality of the lecture | ⑤ ④ ③ ② ① |
| 8 | I am generally satisfied with the speed of the lecture | ⑤ ④ ③ ② ① |
| 9 | Feedback via email was done properly | ⑤ ④ ③ ② ① |

10. Please indicate your preference for online or offline class.

① Strongly prefer offline ② Slightly prefer offline ③ No preference

④ Slightly prefer online ⑤ Strongly prefer online

11. Please indicate your preference for live streaming or recorded video.

① Strongly prefer offline ② Slightly prefer live streaming ③ No preference

④ Slightly prefer recorded video ⑤ Strongly prefer recorded video

■ Please answer each question using 5-point Likert scale

Rating system: 5: strongly agree, 4: agree, 3: neither agree nor disagree, 2: disagree, and 1: strongly disagree.

| I was satisfied with the following strength of online learning | | |
| --- | --- | --- |
| 12 | Taking the course at any time | ⑤ ④ ③ ② ① |
| 13 | Taking the course anywhere | ⑤ ④ ③ ② ① |
| 14 | Flexibility in the sequence of the lecture | ⑤ ④ ③ ② ① |
| 15 | Playing the lecture at any speed they want | ⑤ ④ ③ ② ① |
| 16 | Reviewing multiple times any portion of the lecture | ⑤ ④ ③ ② ① |

| I was dissatisfied with the following weakness of online learning | | |
| --- | --- | --- |
| 17 | Lack of interaction between professor and student | ⑤ ④ ③ ② ① |
| 18 | Lack of interaction among students | ⑤ ④ ③ ② ① |
| 19 | Difficulty in concentrating during online lectures | ⑤ ④ ③ ② ① |
| 20 | Difficulty in maintaining self-directed learning | ⑤ ④ ③ ② ① |

21. Please indicate your opinion on the future direction

① I want most lectures to go back to offline class

② I want online lectures and offline lectures to be properly combined

③ I want most lectures to stay online class

22. Free comments

**Professor Course Evaluation Form**

■ Please answer each question using 5-point Likert scale

Rating system: 5: strongly agree, 4: agree, 3: neither agree nor disagree, 2: disagree, and 1: strongly disagree.

| 1 | Guidance on online training was appropriate and easy to understand | ⑤ ④ ③ ② ① |
| --- | --- | --- |
| 2 | Online teaching (making a lecture video clip or conducting a live online class) was easy | ⑤ ④ ③ ② ① |
| 3 | The environment for making the lecture clip was satisfactory | ⑤ ④ ③ ② ① |
| 4 | There was no inconvenience in booking the place to make the lecture clips | ⑤ ④ ③ ② ① |
| 5 | I am satisfied with the QnA^*^ process after class | ⑤ ④ ③ ② ① |

*QnA: question and answer

6. Please indicate your preference for online or offline class.

① Strongly prefer offline ② Slightly prefer offline ③ No preference

④ Slightly prefer online ⑤ Strongly prefer online

7. Please indicate your preference for live streaming or recorded video.

① Strongly prefer offline ② Slightly prefer live streaming ③ No preference

④ Slightly prefer recorded video ⑤ Strongly prefer recorded video

■ Please answer each question using 5-point Likert scale

Rating system: 5: strongly agree, 4: agree, 3: neither agree nor disagree, 2: disagree, and 1: strongly disagree.

| I was satisfied with the following strength of online learning | | |
| --- | --- | --- |
| 8 | Giving the lecture at any time | ⑤ ④ ③ ② ① |
| 9 | Giving the lecture anywhere | ⑤ ④ ③ ② ① |
| 10 | Correcting the part of the lecture flexibly | ⑤ ④ ③ ② ① |
| 11 | Using the given class time more efficiently | ⑤ ④ ③ ② ① |

| I was dissatisfied with the following weakness of online learning | | |
| --- | --- | --- |
| 12 | Taking more time and effort to prepare for the online lecture | ⑤ ④ ③ ② ① |
| 13 | Copyright issues make it difficult to prepare lecture materials | ⑤ ④ ③ ② ① |
| 14 | The computers and related equipment for online lectures are unfamiliar | ⑤ ④ ③ ② ① |
| 15 | Difficulty in grasping the students’ level of understanding | ⑤ ④ ③ ② ① |
| 16 | Lack of interaction between professor and student | ⑤ ④ ③ ② ① |

17. Please indicate your opinion on the future direction

① I want most lectures to go back to offline class

② I want online lectures and offline lectures to be properly combined

③ I want most lectures to stay online class

18. Free comments
